# Vaccination Against *Lawsonia intracellularis* Decreases Shedding of *Salmonella enterica* serovar Typhimurium in Co-Infected Pigs and Alters the Gut Microbiome

**DOI:** 10.1038/s41598-018-21255-7

**Published:** 2018-02-12

**Authors:** Fernando L. L. Leite, Randall S. Singer, Tonya Ward, Connie J. Gebhart, Richard E. Isaacson

**Affiliations:** 1 Department of Veterinary and Biomedical Sciences, University of Minnesota, St. Paul, MN USA; 2 Biotechnology Institute, University of Minnesota, St. Paul, MN USA

## Abstract

*Salmonella enterica* serovar Typhimurium continues to be a major cause of foodborne illness worldwide and pork can serve as a source of infection. Co-infection of *S. enterica* with *Lawsonia intracellularis*, a common intestinal pathogen of swine, has been found as risk factor for increased *S. enterica* shedding. The objective of this study was to investigate if vaccination against *L. intracellularis* could lead to decreased *S*. Typhimurium shedding. To test this hypothesis, pigs were challenged with either *S*. Typhimurium or *S*. Typhimurium and *L. intracellularis*, with and without *L. intracellularis* vaccination (n = 9 per group). A non-challenged group served as a negative control. Vaccination decreased the shedding of *S*. Typhimurium in co-infected animals by 2.12 log_10_ organisms per gram of feces at 7 days post infection. Analysis of the microbiome showed that vaccination led to changes in the abundance of *Clostridium* species, including *Clostridium butyricum*, in addition to other compositional changes that may explain the protection mediated against *S*. Typhimurium. These results indicate that vaccination against *L. intracellularis* in co-infected herds may provide a new tool to increase food safety by helping to prevent *S. enterica* without the need for antibiotics.

## Introduction

*Salmonella enterica* is a leading cause of foodborne illness worldwide^1^. In the United States, the Centers for Disease Control and Prevention has estimated that *S. enterica* is responsible for over 1 million cases of foodborne illness per year, and is the leading cause of death due to foodborne illnesses^2^. It has been estimated that the economic losses due to salmonellosis in the United States exceed $3 billion per year^3^.

Past studies have estimated that levels of human salmonellosis due to pork consumption vary; in the United States around 6% of outbreaks can be attributable to pork while in western European countries pork has been found to account for 44% of cases^4^. Pigs frequently carry *S. enterica*, and efforts in the United States to reduce the incidence of salmonellosis have mainly remained ineffective. The prevalence of *S. enterica* in pigs is approximately 52.6%^5^ and the incidence of salmonellosis in humans has not been reduced appreciably since 1996^6^. Some believe that control of *S. enterica* in swine may not be possible using current technologies, there are many sources of infection including feed, the environment, and carrier animals^7,8^.

Factors such as stresses associated with transportation to slaughter plants, pathogen transmission during the comingling of pigs during lairage at processing plants, and introduction of new gilts into breeding herds contribute to increased shedding and transmission of *S. enterica* during pork production^9–11^. However, these are not particularly amenable targets for developing improved control strategies. The source of *S. enterica* that contaminates pork products are the animals themselves^10^ and novel intervention strategies are needed.

*L. intracellularis* is a common porcine intestinal pathogen and is prevalent in pig production sites worldwide, with prevalence ranging from 48 to 100% in different swine producing countries^12^. In the United States, it has been estimated that *L. intracellularis* is present in more than 90% of swine farms^13^. *L. intracellularis* causes porcine proliferative enteropathy (PPE) which more commonly occurs in post-weaned pigs and leads to decreased weight gain, diarrhea and is often subclinical. Transmission of this organism also occurs by the fecal-oral route and lesions are marked by a thickening of the mucosa of the ileum and colon^14^.

With the *S. enterica* herd prevalence of 52.6% in US swine, it is reasonable to assume that co-infection with both pathogens occurs frequently. The association between *L. intracellularis* infection and increased shedding of *S. enterica* was first demonstrated by Beloeil *et al*.^15^, who performed an epidemiological study and found a statistically significant association between seroconversion to *L. intracellularis* and increased prevalence of pigs shedding *S. enterica*. Furthermore, we have observed that experimental co-infection of pigs with *L. intracellularis* and *S*. Typhimurium leads to increased colonization of pigs by *S*. Typhimurium^16^. Because of this association between *L. intracellularis* infection and *S. enterica*, we hypothesized that vaccination against *L. intracellularis* would decrease shedding of *S. enterica* in co-infected animals and thus potentially become a new tool to aid in controlling *S*. *enterica* shedding. The importance of the gut microbiome has been established for the maintenance of health, as well as colonization resistance to *S. enterica*^17,18^, and it is known that both *S. enterica* and *L. intracellularis* can alter the composition of the gut microbiome^18,19^. Thus, we further investigated the swine gut microbiome to understand how vaccination and co-infection with *L. intracellularis* may disfavor or favor *S. enterica* colonization, respectively.

## Materials and Methods

### Bacterial challenge inoculum preparation

The nalidixic acid resistant *S. enterica* serovar Typhimurium strain 798 was used for challenge and prepared by overnight growth in Luria-Bertani (LB) broth^19^. The PHE/MN1-00 strain of *L. intracellularis* was grown in McCoy cells for challenge of animals following previously a described protocol^20^.

### Animals and experimental design

The animal protocol #1411-31993A used was approved by the University of Minnesota Institutional Animal Care and Use Committee and all experiments were performed in accordance with relevant guidelines and regulations. In this study, a total of five treatment groups were used: (1) challenged with *S*. Typhimurium alone (Sal), (2) challenged with both *S*. Typhimurium and *L. intracellularis* (Sal Law), (3) challenged with *S*. Typhimurium and vaccinated against *L. intracellularis* (Sal Vac), (4) challenged with both *S*. Typhimurium and *L. intracellularis* and vaccinated against *L. intracellularis* (Sal Law Vac), and (5) non-infected control (Control). To minimize potential differences in microbiome composition prior to the start of the study, animals were obtained from the same herd and co-housed prior to the enrollment in the study. Additionally, animals were randomized in to treatment groups to minimize any potential confounding factors of sex or of which sow gave birth to them. Each group was comprised of nine pigs divided into three separate BSL2 large animal isolation rooms with three animals per room (Supplementary Fig. S1). The non-challenged control group was comprised of six animals divided into two rooms with three pigs per room. These rooms were distributed between two isolation buildings. The groups that were vaccinated against *L. intracellularis* received a single oral dose of a live attenuated vaccine (Enterisol Ileitis, Boehringer Ingelheim) at three weeks of age. Twenty-one days post vaccination, two groups of pigs were challenged with a pure culture of 2 × 10^9^ *L. intracellularis* (strain PHE/MN1-00) organisms per pig. One week post challenge with *L. intracellularis*, all pigs except the non-challenge controls were challenged orally with 1 × 10^8^ *S*. Typhimurium (strain 798) organisms per pig. Fecal samples from pigs were obtained on the day of *L. intracellularis* challenge, on the day of *S*. Typhimurium challenge, two days post *S*. Typhimurium challenge and weekly thereafter until 49 days post challenge with *S*. Typhimurium.

### *S. enterica* quantification

To quantify the amount of *S. enterica* shed in feces of animals, a most probable number enrichment method was used as previously described^21,22^. Briefly, one gram of feces was suspended in 9 ml tetrathionate broth (TTB), diluted in triplicate five-fold dilutions and incubated at 41 °C for 48 hours. One hundred μl of each dilution was then transferred to 900 μl Rappaport-Vassiliadis R10 broth and incubated for 24 hours at 41 °C. Cultured dilutions were then inoculated onto XLT4 agar plates containing 100 μg/ml of Nalidixic Acid (NA) to quantify the challenge strain which was NA resistant. A duplicate inoculation was performed onto XLT4 agar without NA to quantify any other indigenous *S. enterica* strains the pigs could harbor. Colonies with typical *S. enterica* morphology were confirmed by PCR using primers specific for the gene *invA* (Forward Primer: ACAGTGCTCGTTTACGACCTGAAT and Reverse Primer: AGACGACTGGTACTGATCGATAAT)^23^.

### Efficacy of vaccination and *L. intracellularis* infection

To assess the efficacy of vaccination and confirm infection with *L. intracellularis*, antibodies against *L. intracellularis* were investigated in serum samples of animals. Blood samples were collected at different timepoints of the study and analyzed with the immunoperoxidase monolayer assay^24^.

### DNA extraction and 16 S sequencing

For microbiome analysis, DNA was extracted from fecal samples using the MoBio PowerSoil DNA extraction kit. DNA quantity and 260/280 ratios were assessed by Nanodrop. The V1–V3 region of the 16 SrRNA gene was amplified following a dual indexing approach^25^. Primers Meta_V1_27F: TCGTCGGCAGCGTCAGATGTGTATAAGAGACAGAGAGTTTGATCMTGGCTCAG and Meta_V3_534R: GTCTCGTGGGCTCGGAGATGTGTATAAGAGACAGATTACCGCGGCTGCTGG were used in an initial reaction to amplify the V1–V3 variable region followed by a second PCR reaction to add index and flowcell adapters. Both reactions used the KAPA HiFidelity Hot Start Polymerase. Sequencing was performed using the Illumina MiSeq with paired end 300 base pair reads at the University of Minnesota Genomics Center. DNA sequence data is available at the University of Minnesota Digital Conservancy (conservancy.umn.edu).

### Microbiome data processing and analysis

Paired end sequences were quality filtered with trimmomatic^26^ and assembled using FLASH^27^. Homopolymers and ambiguous sequences were removed with the trim.seqs command of Mothur^28^ and chimeras were removed with usearch61 in QIIME (version 1.9.1)^29^. Closed reference operational taxonomic unit (OTU) picking was performed with NINJA^30^ and sequences that did not map to the Greengenes database (version 13_8) underwent de novo picking and assigned taxonomy using uclust^31^ in QIIME. OTUs identified using closed-reference picking and de novo OTUs assigned to 16S taxonomy were combined into one OTU table for analysis. Low abundance and rare OTUs (OTUs present in less than 3 samples and with less than 5 total counts per time point) were filtered out. Samples were rarefied to 9000 sequences per sample to calculate alpha and beta diversity indices. Weighted UniFrac distance^32^ was used to evaluate beta diversity differences between samples. The Chao1 index was used to estimate alpha diversity richness and the Simpson index to evaluate evenness in addition to richness. The Wilcoxon Rank Sum test was used to assess significant differences between treatments as well as Analysis-of-similarities (ANOSIM). For differential abundance testing, DESeq 2 was used with the phyloseq package (version 1.19.1) in R^33^, using a non-rarefied OTU table with samples having less than 7,000 sequences being discarded. DESeq 2 was used as it has been found to be the most sensitive method of differential abundance testing with small sample sizes^34^. OTUs with differential abundance resulting from pairwise comparisons with *p* values below 0.05 corrected for multiple comparisons are reported. The Spearman’s rank correlation was used to test for correlation between OTUs of interest and the level of *S*. Typhimurium shed by animals.

### Statistical analysis

To test for differences in shedding of *S*. Typhimurium between treatments over time, most probable number (MPN) quantification values were used in a linear mixed model with log_10_ MPN as the response, treatment, day, and the treatment/day interaction as fixed effects, barn as a fixed block effect, and pen, pig, and day within pen as random effects. Reported are the least square means for treatment by day, and pairwise comparisons between treatments for each day, with p-values corrected for multiple comparisons. To test for differences in the number of animals shedding per group, the N-1 variation of the Chi-square test was used.

## Results

### *L. intracellularis* vaccination reduces *S*. Typhimurium shedding in co-infected animals

**Figure 1:**
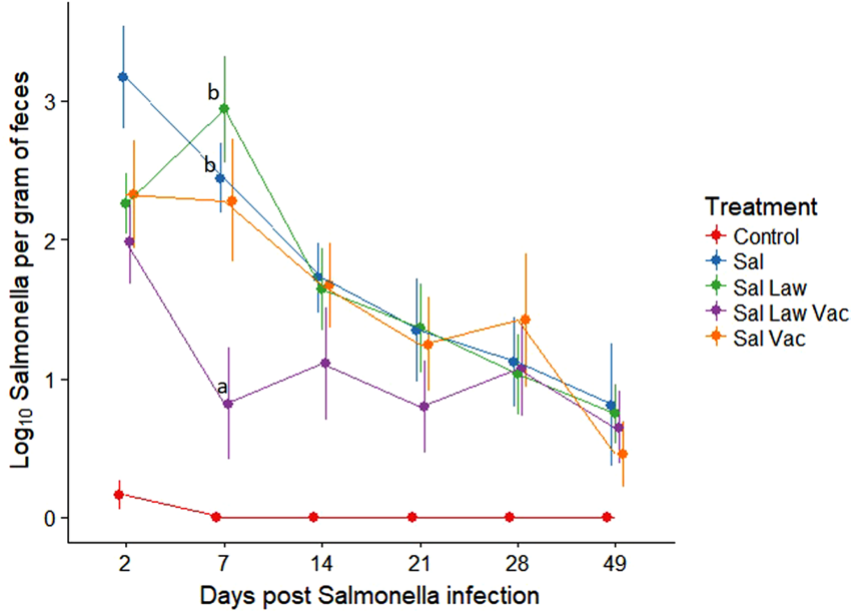
Fecal shedding of *Salmonella enterica* serovar Typhimurium over time, measured by the MPN method. Significant differences between treatment groups are designated by different letters (linear mixed model, error bars represent standard error of the mean *p* < 0.05).

The greatest difference in shedding level between groups was found at 7 days post challenge with *S*. Typhimurium. At this time point, the Sal Law group shed 2.94 log_10_ *S*. Typhimurium organisms per gram of feces, while the Sal Law Vac group shed 0.82 log_10_ *S*. Typhimurium organisms per gram of feces (*p* = 0.003, Fig. 1). The Sal Law Vac group also shed significantly less *S*. Typhimurium then the Sal group, which shed 2.44 Log_10_ *S*. Typhimurium organisms per gram (*p* = 0.03). *L. intracellularis* vaccination did not have a significant impact on *S*. Typhimurium shedding when animals were singly challenged with *S*. Typhimurium. Although not statistically significant, the co-infected vaccinated group was the group that shed the least amount of *S*. Typhimurium at the timepoints of 2, 7, 14 and 21 days post challenge (Fig. 1).

There were significantly less pigs actively shedding *S*. Typhimurium at seven days post challenge in the Sal Law Vac group compared to the other challenged groups (Table 1). Only 38% of pigs shed *S*. Typhimurium in the Sal Law Vac group (*p* < 0.05) compared to 100% of pigs shedding *S*. Typhimurium in all other challenged groups. The trend of having less positive animals in the Sal Law Vac group persisted at other time points, although the differences were not always statistically significant. At the last time point of the study (49 dpi), all challenged groups had a similar number of animals shedding *S*. Typhimurium (from 40% to 50%) and a similar quantity of *S*. Typhimurium.

**Table 1:** Number of animals shedding *S*. Typhimurium at different time points.

Treatment	2 dpi	7 dpi	14 dpi	21 dpi	28 dpi	49 dpi
Control	33% (2/6)^a^	0% (0/6)^a^	0% (0/6)^a^	0% (0/6)^a^	0% (0/6)^a^	0% (0/6)^a^
Sal	100% (9/9)	100% (9/9)^b^	89% (8/9)	67% (6/9)^b,c^	78% (7/9)	44% (4/9)^a,b^
Sal Vac	100% (9/9)	100% (9/9)^b^	89% (8/9)	78% (7/9)^b,c^	67% (6/9)	44% (4/9)^a,b^
Sal Law	100% (9/9)	100% (9/9)^b^	89% (8/9)	100% (9/9)^b^	78% (7/9)	56% (5/9)^b^
Sal Law Vac	100% (8/8)	38% (3/8)^a^	75% (6/8)	50% (4/8)^c^	75% (6/8)	50% (4/8)^b^

Different superscript letters indicate statistical significance (N-1 Variation Chi-Square Test *p* < 0.05). dpi = Days post *S*. Typhimurium infection.

To control for the efficacy of vaccination and of *L. intracellularis* infection, antibodies against *L. intracellularis* were measured in serum of animals. Animals in the Sal Law Vac group had an earlier seroconversion as compared to animals in the Sal Law group, and more animals were positive for antibodies in serum at earlier timepoints in the Sal Law Vac group (4 of 5 animals tested) compared to the Sal Law group (2 of 6 animals tested). At the last timepoint of the study both groups had similar number of animals with anti *L. intracellularis* antibodies in serum (5 of 5 in Sal Law Vac group, 5 of 6 in Sal Law group). There were also animals that developed antibodies in the Sal Vac group demonstrating that the vaccine was effective in eliciting an immune response in the animals. No animals in the Sal and Control groups had antibodies in serum against *L. intracellularis*.

### Changes in microbial community structure associated with co-infection and *L. intracellularis* vaccination

**Figure 2:**
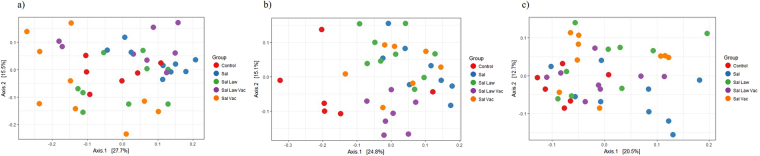
Principal coordinate analysis plot of weighted UniFrac distance among different treatment groups. (**a**) 0 days post *S*. Typhimurium challenge; (**b**) 7 days post *S*. Typhimurium challenge; (**c**) 14 days post *S*. Typhimurium challenge.

Previous studies showed that oral challenge with either *S*. Typhiumrium or *L. intracellularis* led to alterations in the composition of the swine gut microbiome^19,35,36^. To determine if vaccination with the live oral attenuated vaccine against *L. intracellularis* would lead to changes in the microbiome, we looked for community composition differences between treatment groups using the weighted UniFrac distance^32^. At 7 days post challenge with *S*. Typhimurium, which was when the greatest difference in *S*. Typhimurium shedding between groups was observed, treatment had a significant impact on the grouping of samples as confirmed by ANOSIM (*p* = 0.001, Fig. 2b). The principal coordinate plot (PCoA), shows that the Sal Law Vac group mainly clustered apart from all other treatment groups. This effect was dependent on animals receiving both *S*. Typhimurium and *L. intracellularis* challenge as well as the *L. intracellularis* vaccine (Fig. 2b). This clustering pattern was not observed prior to *S*. Typhimurium challenge (Fig. 2a) or 14 days post challenge with *S*. Typhimurium (Fig. 2c).

**Figure 3:**
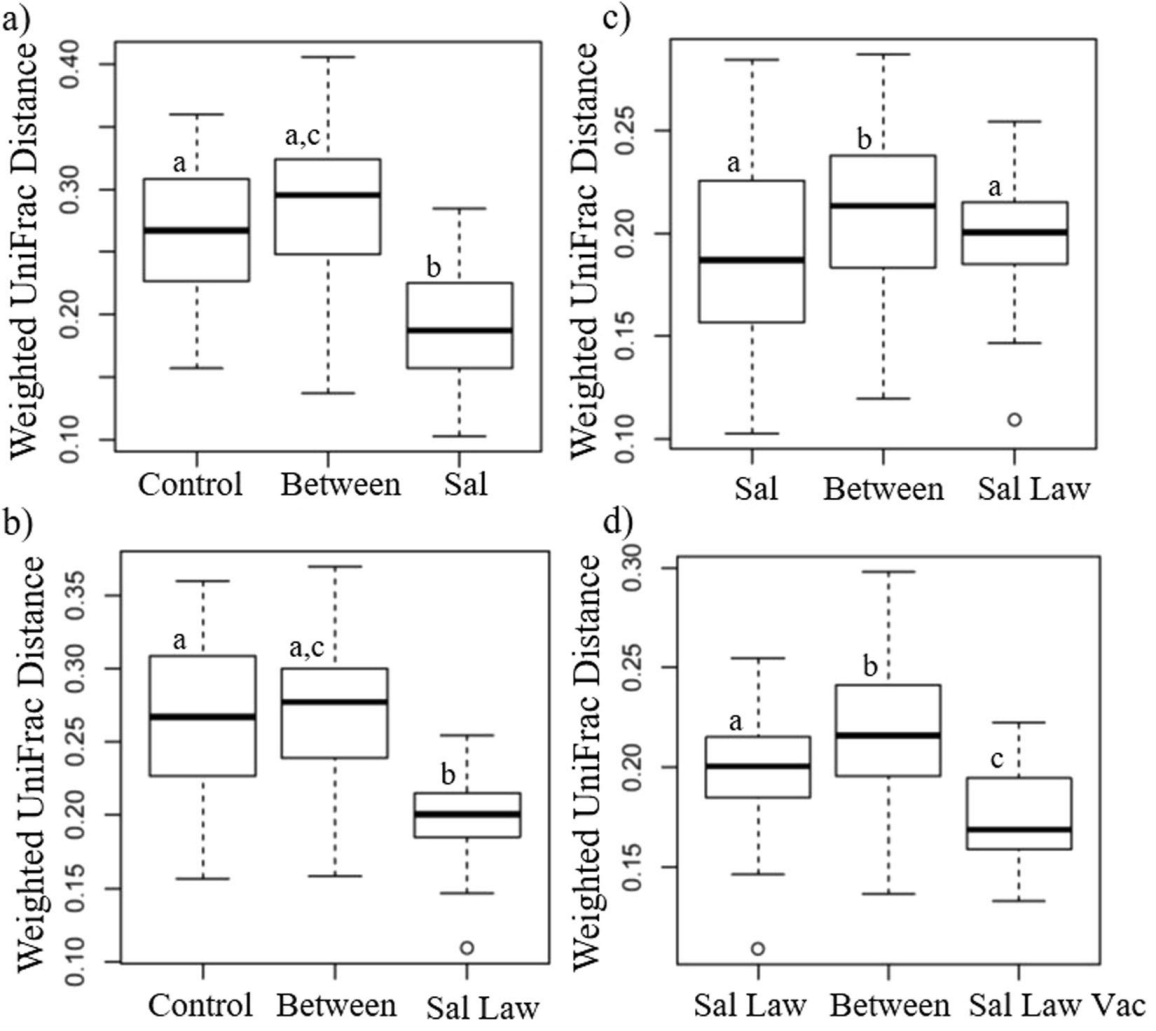
Weighted UniFrac distance within and between treatments at 7 days post *S*. Typhimurium infection. Between is the distance between all samples in the comparison. (**a**) Control non-infected compared to single *S*. Typhimurium infection; (**b**) Control non-infected compared to dual *S*. Typhimurium, *L. intracellularis* infection; (**c**) Single *S*. Typhimurium infection compared to dual S. Typhimurium, *L. intracellularis* infection; (**d**) Dual *S*. Typhimurium, *L. intracellularis* infection compared to dual infection with previous *L. intracellularis* vaccination. Different letters indicate statistical significance (Wilcoxon Rank Sum Test *p* < 0.05).

*S*. Typhimurium challenge in the Sal group was associated with a convergence of microbiome composition on day 7 post challenge in comparison to non-infected control animals (Fig. 3a). No significant difference was found between these two groups prior to challenge, or at 14 days post challenge (Supplementary Fig. S2). Prior to challenge with *S*. Typhimurium, the Sal Law group, which had only received *L. intracellularis* challenge, was significantly more variable than the non-infected control group (Supplementary Fig. S3), but following *S*. Typhimurium challenge their microbiomes also converged to a more similar composition compared to control non-infected animals (Fig. 3b). No significant difference was found comparing the weighted UniFrac distance within singly (Sal) and dually (Sal Law) infected animals (Fig. 3c).

Vaccinated and dually infected animals had significantly less variable microbial communities than those dually infected and not vaccinated (Fig. 3d). This demonstrates that animals that received the vaccine responded more similarly to each other than those that received co-challenge without vaccination. This highlights the effect of the vaccine and suggests animals in that group responded in a similar fashion, which is also demonstrated by a distinct clustering pattern observed with this group in the PCoA (Fig. 2b). Of note, enhanced similarity between the microbiomes of vaccinated and dually challenged (Sal Law Vac) animals compared to dually challenged animals without vaccination (Sal Law) persisted until 21 days post *S*. Typhimurium challenge (Supplementary Fig. S4). Comparing singly infected (Sal) to singly infected and vaccinated animals (Sal Vac), no differences in community composition were found within the two groups (Supplementary Fig. S5), demonstrating that the effect of vaccination is different with and without subsequent *L. intracellularis* challenge.

**Figure 4:**
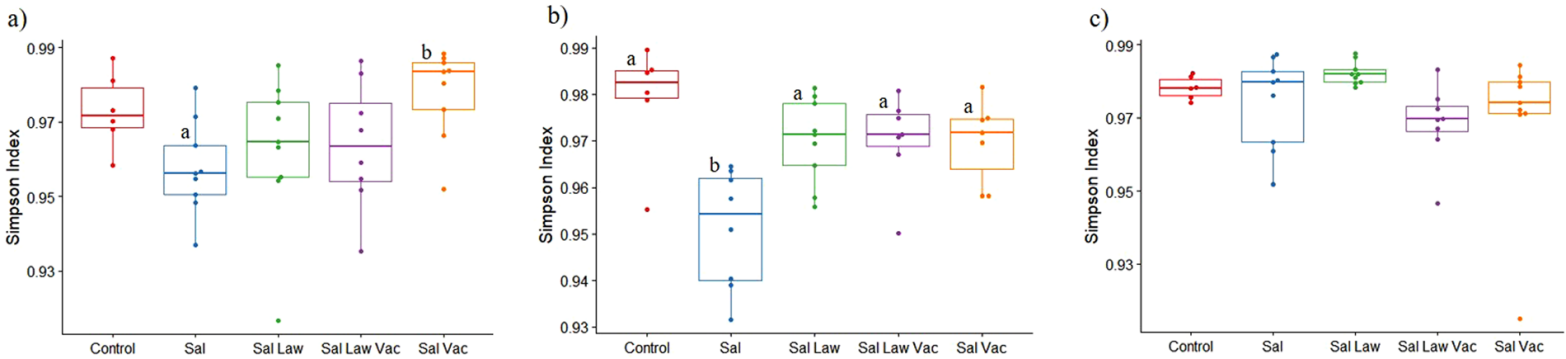
Simpson index among different treatment groups at (**a**) 0 days post *S*. Typhimurium infection; (**b**) 7 days post *S*. Typhimurium infection; (**c**) 49 days post *S*. Typhimurium infection. Different letters indicate statistical significance (Wilcoxon Rank Sum Test *p* < 0.05).

No statistical differences were observed with the Chao1 estimate of richness or in the number of observed OTUs between treatments (also an estimate of richness) (Supplementary Fig. S6). The Simpson index, which takes into account evenness and richness within a sample, did show significant differences between treatments. Prior to challenge with *S*. Typhimurium, the only statistically significant difference was between the Sal and the Sal Vac group (Fig. 4a). At seven days post-challenge with *S*. Typhimurium, all treatment groups that received *L. intracellularis* had a significantly higher Simpson index compared to the Sal group (Fig. 4b). This suggests that exposure to *L. intracellularis* either through challenge or attenuated live vaccine impacts the composition of the microbiome in response to *S*. Typhimurium infection in a similar manner in terms of maintaining diversity of taxa as measured by the Simpson index. At 14 days post infection, no statistical differences were observed between any of the treatments and at the last time point of the study (49 days post *S*. Typhimurium infection) all groups had very similar Simpson indices (Fig. 4c).

### Phylogenetic differences observed in the microbiome associated with challenge and *L. intracellularis* vaccination

**Figure 5:**
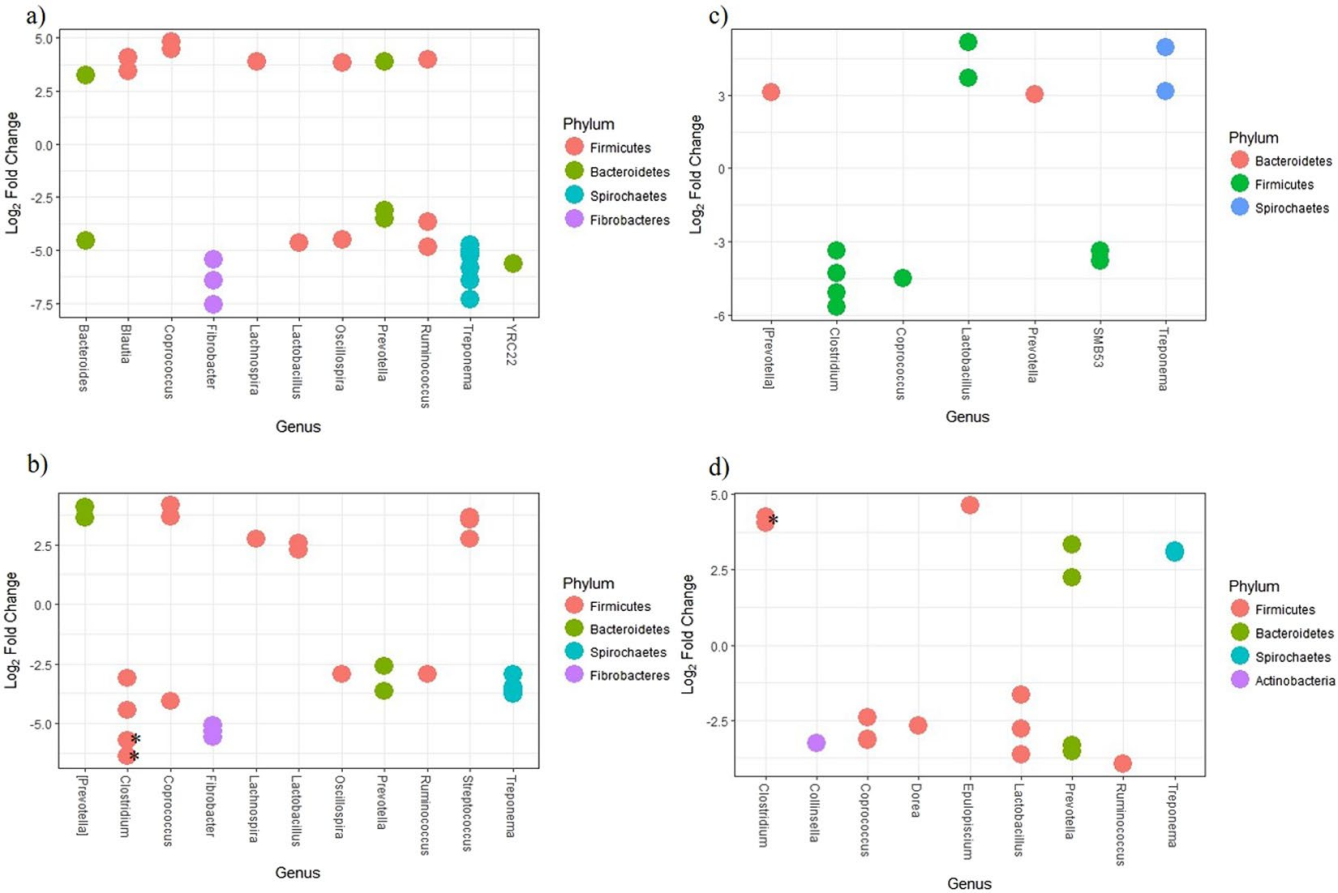
Differentially abundant bacteria identified comparing different treatments at 7 days post *S*. Typhimurium infection. Each dot represents a different OTU identified within a genus. (**a**) Control compared to Sal, a positive log fold change indicates enrichment in the Sal group. (**b**) Control compared to Sal Law, a positive log fold change indicates enrichment in the Sal Law group; (**c**) Sal compared to Sal Law, a positive log fold change indicates enrichment in the Sal Law group; (**d**) Sal Law compared Sal Law Vac, a positive log fold change indicates enrichment in the Sal Law Vac group. *OTU sequences closely related to *Clostridium butyricum*.

As expected, the swine gut microbiome was dominated by the phyla Bacteroidetes and Firmicutes (relative abundance of both phyla ranged from 71% to 87% among different treatments and timepoints, Supplementary Fig. S7)^37^. The succession of the swine microbiome is well known^38^, therefore we analyzed the effects of each treatment within each time point separately. Two of the six animals in the Control group shed *S*. Typhimurium at 2 days post infection (dpi), these animals were still considered in microbiome analysis since they shed very low levels and at only one of the several timepoints of the study. We additionally analyzed data with and without these animals and saw no differences (data not shown). To investigate compositional differences in the microbiome that may have led to the observed reduction in shedding of *S*. Typhimurium due to *L. intracellularis* vaccination, we focused on the time point of seven days post challenge with *S*. Typhimurium, when *L. intracellularis* vaccination led to the greatest reduction in shedding. At this time point, several significant (*p* < 0.05) differences in the abundance of OTUs at the genus level were observed comparing the different treatment groups (Fig. 5). This was also the time point with the greatest number of significantly differently abundant OTUs between the Sal Law Vac and Sal Law group.

At seven days post *S*. Typhimurium challenge, the Sal group compared to Controls had a significant (*p* < 0.05) increase in the abundance of the genera *Coprococcus, Lachnospira* and *Blautia* and a decrease in *Lactobacillus*, *Treponema*, *Fibrobacter* and YCR22 (Fig. 5a). OTUs belonging to 4 different genera (*Ruminococcus*, *Prevotella*, *Oscillospira* and *Bacteoides*) had both a significant (*p* < 0.05) increase and decrease in abundance in the Sal group compared to the Control group (Fig. 5a). One of the OTUs of the *Prevotella* genus that was enriched in the Sal group compared to Control was closely related to the *Prevotella copri* species. None of the differences observed between these two groups were observed prior to challenge, except for differential abundance of *Lachnospira* and *Oscillospira* which may have been due to stochastic randomness when animals were divided in to groups (Supplementary Fig. S8a).

Pigs in both the Sal and Sal Law groups had significant decreases of *Treponema* and *Fibrobacter* (*p* < 0.05) and a significant increase in *Lachnospira* compared to Controls (*p* < 0.05, Fig. 5a,b). Dual challenge differed in that the Sal Law group had a significant decrease in the abundance of *Clostridium* (*p* < 0.05) and a significant enrichment of *Streptococcus* and *Lactobacillus*, which was not observed with the Sal group compared to Controls (*p* < 0.05, Fig. 5a,b). Two of the OTUs of the *Clostridium* genus that were significantly reduced in abundance in the Sal Law group were closely related to the species *C. butyricum*. Similar to the effect of the Sal Law group compared to Controls, comparing the Sal group to the Sal Law group, the Sal Law group had a significant higher abundance of *Lactobacillus*, an increase of [*Prevotella*] and a decrease in the abundance of *Clostridium* (*p* < 0.05, Fig. 5c). The designation of [*Prevotella*] was given because its taxonomy is unknown but predicted to be *Prevotella*. Since these changes were observed when comparing the Sal Law group to Controls as well as the Sal Law group to the Sal group, they were likely mediated by *L. intracellularis* and due to dual challenge. Analyzing the microbiome of single *L. intracellularis* infection prior to *S*. Typhimurium infection confirmed that *L. intracellularis* alone can lead to decreased abundance of *Clostridium* and sequences that closely relate to *C. butyricum* as well as an increase in abundance of *Lactobacillus* compared to non-infected animals (Supplementary Fig. S9), these differences in abundance were not observed prior to *L. intracellularis* challenge.

Comparing vaccinated dually challenged (Sal Law Vac) animals to non-vaccinated dually challenged animals (Sal Law), the Sal Law Vac group had a significant decrease in abundance of *Lactobacillus* and a significant increase in the abundance of *Clostridium* (*p* < 0.05, Fig. 5d). One of the OTUs within the *Clostridium* genus most closely matched the *C. butyricum* species. The increase of sequences that closely match *C. butyricum* was also mediated by vaccination prior to *S*. Typhimurium challenge when the Sal Law and Sal Law Vac groups had only received *L. intracellularis* challenge (*p* < 0.05, Supplementary Figure S8d), demonstrating that increased abundance of *C. butyricum* is an effect of the vaccine on *L. intracellularis* infection. There was no difference in the abundance of OTUs between these two groups prior to *L. intracellularis* infection (timepoint -7 days post *S*. Typhimurium infection). The abundance of sequences related to *C. butyricum* were significantly negatively correlated to *S*. Typhimurium shedding levels at 7 days post *S*. Typhimurium challenge (Supplementary Table S1).

Further comparing the Sal Law to the Sal Law Vac group at day 7 post *S*. Typhimurium challenge, we found a significant decrease in abundance of an OTU that closely matches *Collinsella aerofaciens*, two OTUs that closely match *Prevotella copri* and another OTU pertaining to the genus *Dorea* (*p* < 0.05) and an enrichment of *Epulopiscium* in the Sal Law Vac group (*p* < 0.05, Fig. 5d). Changes in the abundance of *P. copri* and *C. aerofaciens* were not observed when comparing the effect of vaccination on single *S*. Typhimurium infection (Sal to Sal Vac) or when comparing the effect of co-infection (Sal Law) to non-infected animals (Control), suggesting that these changes are specific to the effect of vaccination on co-challenge (Fig. 5b, Supplementary Fig. S10a). The abundance of sequences related to both *C. aerofaciens* and *P. copri* were significantly (*p* < 0.05) positively correlated to the level of *S*. Typhimurium shedding in pigs at 7 dpi (Supplementary Table S1).

Comparing pigs in the Sal group to those in the Sal Vac group, the live attenuated vaccine was associated with a significant decrease in abundance of sequences related to *Clostridium butyricum* (*p* < 0.05, Supplementary Fig. S10a), further suggesting that differential abundance of *Clostridium* is related to *L. intracellularis* infection. In this group, co-challenge was due to having received the live oral attenuated vaccine. This effect was also observed when analyzing the impact of vaccination alone comparing vaccinated to non-vaccinated animals prior to *S*. Typhimurium infection (Supplementary Fig. S11). Since the Sal Vac group, which had lower levels of *C. butyricum* group did not shed more *S*. Typhimurium than the Sal group which was not exposed to *L. intracellularis* and lower *C. butyricum* levels, differential abundance of *C. butyricum* alone likely is not sufficient to drive a change in *S*. Typhimurium shedding levels.

The only observed significant difference at the genus level between the Sal Law group and the Sal Vac group was a decrease in the abundance of *Treponema* (Supplementary Fig. S10b). This suggests that both groups had similar microbiomes and responded similarly to *S*. Typhimurium challenge. This also is supported by the fact that there were no significant changes in *S*. Typhimurium shedding between both groups (Fig. 1, Table 1) and as noted above, at 7 days post *S*. Typhimurium challenge both groups had similar alpha and beta diversities, as well as similar clustering as visualized by PCoA (Fig. 2b). Thus, compositional and structural changes in the microbiome and reduction in shedding levels of *S*. Typhimurium mediated by *L. intracellularis* vaccination were dependent on immunization and challenge as opposed to vaccination alone.

## Discussion

*S. enterica* remains one of the leading causes of foodborne illness worldwide and improved strategies to reduce the prevalence of *S. enterica* in swine are much needed^1,7^. Previous research has found that *L. intracellularis* can increase the susceptibility of a pig to *S. enterica* and increase its shedding^15,16^. In this study, we found that vaccination against *L. intracellularis* results in a decrease in the shedding of *S. enterica* in co-challenged animals. Other co-infections that favor non-typhoidal *Salmonella* are known to occur, such as co-infection with porcine respiratory and reproductive syndrome virus in swine^39^ and with malaria in humans^40^. Few studies have investigated the impacts of vaccination against pre-disposing organisms to improve the control of *S. enterica* nor have they investigated the underlying changes in the composition of the microbiome that may be associated with increased susceptibility due to co-challenge.

In the study described here, vaccinating pigs against *L. intracellularis* three weeks prior to challenge with *L. intracellularis* decreased the shedding of *S*. Typhimurium by 2.12 log_10_ organisms in dually infected pigs compared to pigs receiving the same co-challenge without vaccination at 7 days post *S*. Typhimurium infection (Fig. 1). Vaccination against *L. intracellularis* also led to a significant reduction in the number of animals shedding *S*. Typhimurium. All groups challenged with *S*. Typhimurium exhibited 100% of pigs shedding *S*. Typhimurium except the group that was vaccinated and co-challenged which only had 38% of pigs shedding *S*. Typhimurium at 7 days post *S*. Typhimurium infection (Table 1). Clinical signs were monitored in this study and while few animals exhibited signs of diarrhea, those with diarrhea were evenly distributed through the challenged treatment groups (data not shown). Thus, subclinical infection was induced in this experiment as is often the presentation of *L. intracellularis* and *S*. Typhimurium infections in the field^14,18^.

The desired properties of a vaccine to protect against *S. enterica* and to improve food safety are to reduce shedding and/or tissue colonization, protect against different serovars, and not interfere with serologic monitoring of *S. enterica* in the herd^41^. It is also advantageous if the vaccine is cost effective. Vaccination against *L. intracellularis* has the inherent advantage of not interfering with the serologic diagnosis of *S. enterica*, and as demonstrated here, also reduces the shedding of *S*. Typhimurium. In herds infected with *L. intracellularis*, this vaccine has been demonstrated to be cost effective, thus protecting against *S. enterica* would only add to its value^42^. Meschede^43^. tested the impact of *L. intracellularis* vaccination in herds that had a high prevalence of both *L. intracellularis* and *S. enterica* infections. The study found a decrease in *S. enterica* prevalence in vaccinated groups as measured by antibody titers in four farms included in their study.

To explore some of the underlying mechanisms of the decrease in *S*. Typhimurium due to *L. intracellularis* vaccination, we investigated the gut microbiome, which is known to be altered by *S. enterica*, to enhance its successful infection of animals^17,18^. We investigated alpha diversity, beta diversity, and compositional differences at the genus/OTU level between treatments. Alpha diversity analysis revealed that significant changes caused by *S*. Typhimurium infection were mainly in evenness and not richness. These results are similar to those of Bearson *et al*.^35^ who also found significant changes only in measures of evenness and not richness in pigs challenged with *S*. Typhimurium. This indicates that the number of species in the gut isn’t impacted as much as their quantities by *S*. Typhimurium infection. With more time post challenge and a decrease in *S*. Typhimurium shedding, the microbiome of the different treatment groups became more similar and at the last time point of the study there were very little differences in alpha diversity as measured by the Simpson index among the microbiomes of the different treatment groups (Fig. 4c).

Using the weighted UniFrac distance to evaluate community structure differences between treatments, it was possible to observe that *S*. Typhimurium had a significant impact on both singly and dually challenged animals and vaccination prior to dual infection also led to a different response from both groups (Fig. 3a,b,d). The dually challenged vaccinated group clustered apart from all other treatment groups at 7 days post challenge, when it led to the greatest decrease in *S*. Typhimurium shedding (Fig. 2b). The vaccinated co-challenged group also had lower within group UniFrac distances compared to those dually infected without vaccination, suggesting that animals responded more similarly to each other if they had received *L. intracellularis* vaccination prior to challenge and had a more similar microbiome.

To further understand how shifts in the microbiome may have mediated the decrease in *S. enterica* shedding, we looked for differences in the abundance of different genera between different treatments. Similar to our previous study investigating the microbiome of single and dual challenge with *L. intracellularis* and *S*. Typhimurium^19^, co-challenge with *L. intracellularis* led to an increase of *Lactobacillus* and a decrease in abundance of *Prevotella* and *Treponema*. In this study, the decrease in abundance of *Treponema* and *Prevotella* was found comparing co-challenged to non-challenged control animals. While co-challenge led to an increase in the abundance of both compared to animals only challenged with *S*. Typhimurium (Fig. 5b,c).

Drumo *et al*.^36^ found that increased abundance of *Lactobacillus* was associated with increased *S*. Typhimurium virulence, shedding and increased inflammation in the gut of pigs. The increased abundance of *Lactobacillus* with co-challenge is further indicative that co-challenge favors *S. enterica* infection. Interestingly, when comparing the Sal Law Vac and Sal Law groups, the Sal Law Vac group had a lower abundance of *Lactobacillus*. This demonstrates that vaccination can alter the composition of the microbiome in response to *S*. Typhimurium in a manner that has been associated with less severe infection, which in our study was associated with decreased shedding.

Multiple studies have observed a decrease in the abundance of *Prevotella* with *S*. Typhimurium infection in swine^19,35,36^. In our study, we found different responses within different OTUs of this genus. Vaccinated co-challenged animals had increased levels of some OTUs of *Prevotella* and a decrease in others compared to co-challenged without vaccination. The two OTUs that decreased in abundance in the Sal Law Vac group closely matched the species *Prevotella copri*. We could not determine the species of the *Prevotella* that increased. Comparing control non-challenged animals to animals only challenged with *S*. Typhimurium, *S*. Typhimurium led to an increase in the abundance of sequences that match *P. copri* and a decrease of two *Prevotella* with unidentified species. *P. copri* has been considered a pathobiont and implicated in chronic inflammatory diseases such as rheumatoid arthritis and has been shown to have a high capacity to induce pro-inflammatory responses in the gut^44,45^. Thus, it potentially could favor *S. enterica* in the establishment of inflammation that is needed for colonization^18^.

Interestingly, another pathobiont species was also decreased by vaccination. *Collinsella aerofaciens* is regarded as the most abundant bacterium in the human gut and some strains have the capacity to induce arthritis and high amounts of pro-inflammatory cytokines due to their cell wall components^46–48^. A significant difference in the abundance of sequences matching *C. aerofaciens* was only found due to vaccination in co-challenged pigs. Interestingly, sequences pertaining to both *P. copri* and *C. aerofaciens* were significantly positively correlated to the level of *S*. Typhimurium shedding (Supplemental Table 1), further suggesting their association with *S*. Typhimurium infection. Other bacteria that were uniquely altered by vaccination in co-challenged pigs were *Dorea* and *Epulopiscium* (Fig. 5d).

In contrast to the possible pro-inflammatory effects of *P. copri* and *C. aerofaciens*, *Clostridium* species have been shown to have a major role in immunosuppression. Through fermentation of dietary fiber, *Clostridium* species produce short-chain fatty acids that are immunosuppressive and help to maintain overall intestinal homeostasis^49^. Among the short chain fatty acids produced by this genus is butyrate which, in addition to having anti-inflammatory properties, is an important nutrient for colonocytes, and can also down regulate the expression of Salmonella Pathogenicity Island 1 (SPI-1) virulence genes of *S*. Typhimurium, which are important for cell invasion^49,50^. Depletion of butyrate producing *Clostridium* also can promote *S. enterica* infection by allowing for aerobic expansion in the gut^51^. In this study, *S*. Typhimurium alone did not cause a disturbance in the abundance of *Clostridium*, however co-challenge led to a decrease in its abundance (Fig. 5b,c). This decrease may be involved in the increased susceptibility to *S*. Typhimurium colonization and shedding mediated by dual challenge with *S*. Typhimurium and *L. intracellularis*. Vaccination against *L. intracellularis* led to an increase in the abundance of *Clostridium* compared to co-infection without vaccination. This increase included a 4.27 log_2_ fold increase in the abundance of sequences that match to *Clostridium butyricum* (Fig. 5d). *C. butyricum* was given its name due to its capacity to produce large amounts of butyrate and non-toxigenic strains are widely used as probiotics in Asia^52^. The decrease of *Clostridium*, and *C. butyricum* in particular, could potentially benefit *S. enterica* serovars other than Typhimurium, since different serovars can have similar mechanisms of infection with the use of SPI-1 genes^18^. Therefore, the increase in *Clostridium* and *C. butyricum* abundance due to *L. intracellularis* vaccination in co-infected animals potentially could reduce the shedding of different serovars, another desired quality of a *S. enterica* vaccine. This possibility should be confirmed with more directed experiments.

This study demonstrated for the first time that vaccination against *L. intracellularis*, a common pathogen in swine, is able to reduce the level and prevalence of *S*. Typhimurium shedding. The effect of vaccination was dependent upon challenge with *L. intracellularis*, which suggests that apart from changes in the microbiome an immune response may also be involved in these observations. The use of vaccination against *L. intracellularis* to control *S. enterica* is a novel and promising new tool that is much needed for controlling *S. enterica* in pig herds as well as improving food safety, and may be an alternative to the use of antimicrobials.

## Electronic supplementary material

Supplementary Information

## References

[1] Kirk MD (2015). World Health Organization Estimates of the Global and Regional Disease Burden of 22 Foodborne Bacterial, Protozoal, and Viral Diseases, 2010: A Data Synthesis. PLoS Med..

[2] Scallan E (2011). Foodborne illness acquired in the United States-Major pathogens. Emerg. Infect. Dis..

[3] Hoffmann S, Batz MB, Morris JG (2012). Annual Cost of Illness and Quality-Adjusted Life Year Losses in the United States Due to 14 Foodborne Pathogens. J. Food Prot..

[4] Pires SM, Vieira AR, Hald T, Cole D (2014). Source Attribution of Human Salmonellosis: An Overview of Methods and Estimates. Foodborne Pathog. Dis..

[5] USDA. Salmonella on U.S. Swine Sites - Prevalence and Antimicrobial Susceptibility. (2009).

[6] Boore AL (2015). Salmonella enterica infections in the United States and assessment of coefficients of variation: A Novel approach to identify epidemiologic characteristics of individual serotypes, 1996–2011. PLoS One.

[7] SA, C., AE, B. & RW, G. Carlson SA Salmonelosis.pdf. in *Diseases of Swine* (eds. Zimmerman, J. J., Karriker, L. A., Ramirez, A. & Schwartz, K. J.) 821–833 (John Wiley & Sons, Inc., 2012).

[8] O’Connor AM, Denagamage T, Sargeant JM, Rajić A, McKean J (2008). Feeding management practices and feed characteristics associated with Salmonella prevalence in live and slaughtered market-weight finisher swine: A systematic review and summation of evidence from 1950 to 2005. Prev. Vet. Med..

[9] Isaacson R, Firkins L, Weigel R, Zuckermann F, DiPietro J (1999). Effect of transportation and feed withdrawal on shedding of Salmonella typhimurium among experimentally infected pigs. Am. J. Vet. Res..

[10] Dickson, J. S. & Hurd, H. S. Salmonella in the Pork Production Chain. *National Pok Board, Pork Safety Fact Sheet* **2015** (2013).

[11] Penmetchsa TV, White BA, Maddox CW, Firkins LD, Weigel RM (2009). Molecular epidemiologic investigation of the role of gilts in the introduction and transmission of Salmonella in swine production systems. J. Swine Heal. Prod..

[12] Dors A, Pomorska-Mól M, Czyzewska E, Wasyl D, Pejsak Z (2015). Prevalence and risk factors for lawsonia intracellularis, brachyspira hyodysenteriae and Salmonella spp. in finishing pigs in Polish farrow-to-finish swine herds. Pol. J. Vet. Sci..

[13] Armbruster, G. A. *et al*. Review of Lawsonia intracellularis seroprevalence screening in the United States, June 2003 to July 2006. In *38th Annual Meeting of the American Association of Swine Veterinarians* 231–234 (2007).

[14] Vannucci FA, Gebhart CJ (2014). Recent Advances in Understanding the Pathogenesis of *Lawsonia intracellularis* Infections. Vet. Pathol..

[15] Belœil PA (2004). Risk factors for Salmonella enterica subsp. enterica shedding by market-age pigs in French farrow-to-finish herds. Prev. Vet. Med..

[16] Isaacson, R., *et al* intracellularis increases Salmonella enterica levels in the intestines of pigs. In *Conference of Research Workers in Animal Diseases* 103 (2011).

[17] Ahmer BMM, Gunn JS (2011). Interaction of Salmonella spp. With the intestinal microbiota. Front. Microbiol..

[18] Kim HB, Isaacson RE (2017). *Salmonella* in Swine: Microbiota Interactions. Annu. Rev. Anim. Biosci..

[19] Borewicz KA (2015). Changes in the porcine intestinal microbiome in response to infection with Salmonella enterica and Lawsonia intracellularis. PLoS One.

[20] Guedes RMC, Gebhart CJ (2003). Onset and duration of fecal shedding, cell-mediated and humoral immune responses in pigs after challenge with a pathogenic isolate or attenuated vaccine strain of Lawsonia intracellularis. Vet. Microbiol..

[21] Davies PR (2000). Comparison of methods for isolating *Salmonella* bacteria from faeces of naturally infected pigs. J. Appl. Microbiol..

[22] Kim HB (2014). Effects of tylosin administration on C-reactive protein concentration and carriage of Salmonella enterica in pigs. Am. J. Vet. Res..

[23] Singer RS, Cooke CL, Maddox CW, Isaacson RE, Wallace RL (2006). Use of pooled samples for the detection of Salmonella in feces by polymerase chain reaction. J. Vet. Diagn. Invest..

[24] Guedes RMC, Gebhart CJ, Deen J, Winkelman NL (2002). Validation of an immunoperoxidase monolayer assay as a serologic test for porcine proliferative enteropathy. J. Vet. Diagn. Invest..

[25] Gohl DM (2016). Systematic improvement of amplicon marker gene methods for increased accuracy in microbiome studies. Nat. Biotechnol..

[26] Bolger AM, Lohse M, Usadel B (2014). Trimmomatic: A flexible trimmer for Illumina sequence data. Bioinformatics.

[27] Magoč T, Salzberg SL (2011). FLASH: Fast length adjustment of short reads to improve genome assemblies. Bioinformatics.

[28] Schloss PD (2009). Introducing mothur: Open-source, platform-independent, community-supported software for describing and comparing microbial communities. Appl. Environ. Microbiol..

[29] Caporaso JG (2010). QIIME allows analysis of high-throughput community sequencing data. Nat. Methods.

[30] Al-Ghalith GA, Montassier E, Ward HN, Knights D (2016). NINJA-OPS: Fast Accurate Marker Gene Alignment Using Concatenated Ribosomes. PLoS Comput. Biol..

[31] Edgar RC (2010). Search and clustering orders of magnitude faster than BLAST. Bioinformatics.

[32] Lozupone CA, Hamady M, Kelley ST, Knight R (2007). Quantitative and Qualitative Diversity Measures Lead to Different Insights into Factors That Structure Microbial Communities. Appl. Environ. Microbiol..

[33] McMurdie, P. J. & Holmes, S. Phyloseq: An R Package for Reproducible Interactive Analysis and Graphics of Microbiome Census Data. *PLoS One* **8** (2013). 10.1371/journal.pone.0061217 PMC3632530 23630581

[34] Weiss S (2017). Normalization and microbial differential abundance strategies depend upon data characteristics. Microbiome.

[35] Bearson SMD (2013). Profiling the gastrointestinal microbiota in response to Salmonella: Low versus high Salmonella shedding in the natural porcine host. Infect. Genet. Evol..

[36] Drumo R (2016). Salmonella enterica Serovar Typhimurium Exploits Inflammation to Modify Swine Intestinal Microbiota. Front. Cell. Infect. Microbiol..

[37] Isaacson R, Kim HB (2012). The intestinal microbiome of the pig. Anim. Heal. Res. Rev..

[38] Kim Y, Mylonakis E (2011). Killing of Candida albicans filaments by Salmonella enterica serovar Typhimurium is mediated by sopB effectors, parts of a type III secretion system. Eukaryot. Cell.

[39] Boyen F (2008). Non-typhoidal Salmonella infections in pigs: A closer look at epidemiology, pathogenesis and control. Vet. Microbiol..

[40] Takem E, Roca A, Cunnington A (2014). The association between malaria and non-typhoid Salmonella bacteraemia in children in sub-Saharan Africa: a literature review. Malar. J..

[41] Wales AD, Davies RH (2017). Salmonella Vaccination in Pigs: A Review. Zoonoses Public Health.

[42] Park S (2013). Efficacy of a commercial live attenuated Lawsonia intracellularis vaccine in a large scale field trial in Korea. Clin. Exp. Vaccine Res..

[43] Meschede J (2014). Reduzierung der Salmonellenprävalenz in Lawsonia intracellularis- infizierten Schweinebeständen mittels Enterisol® Ileitis-lmpfung. Prakt. Tierarzt.

[44] Dillon SM (2016). Gut dendritic cell activation links an altered colonic microbiome to mucosal and systemic T-cell activation in untreated HIV-1 infection. Mucosal Immunol..

[45] Maeda, Y. *et al*. Dysbiosis Contributes to Arthritis Development via Activation of Autoreactive T Cells in the Intestine. **68**, 2646–2661 (2016). 10.1002/art.39783 27333153

[46] Kageyama A, Sakamoto M, Benno Y (2000). Rapid identification and quantification of Collinsella aerofaciens using PCR. FEMS Microbiol. Lett..

[47] Zhang, X., Rimpilainen, M., Simelyte, E. & Toivanen, P. Enzyme Degradation and Proinflammatory Activity in Arthritogenic and Nonarthritogenic Eubacterium aerofaciens Cell Walls. **69**, 7277–7284 (2001). 10.1128/IAI.69.12.7277-7284.2001 PMC98812 11705898

[48] Chen, J. *et al*. An expansion of rare lineage intestinal microbes characterizes rheumatoid arthritis. *Genome Med*. 1–14, 10.1186/s13073-016-0299-7 (2016). 10.1186/s13073-016-0299-7 PMC4840970 27102666

[49] Blander JM, Longman RS, Iliev ID, Sonnenberg GF, Artis D (2017). Regulation of inflammation by microbiota interactions with the host. Nat. Immunol..

[50] Gantois I (2006). Butyrate Specifically Down-Regulates Salmonella Pathogenicity Island 1. Gene Expression..

[51] Zhang, L. F. *et al*. Depletion of Butyrate-Producing Clostridia from the Gut Microbiota Drives an Aerobic Luminal Expansion of Salmonella Article Depletion of Butyrate-Producing Clostridia from the Gut Microbiota Drives an Aerobic Luminal Expansion of Salmonella. 443–454, 10.1016/j.chom.2016.03.004 (2016). 10.1016/j.chom.2016.03.004 PMC4832419 27078066

[52] Cassir N, Benamar S, La Scola B (2016). Clostridium butyricum: From beneficial to a new emerging pathogen. Clin. Microbiol. Infect..

