# Supplementary material for: Vaccination Against Lawsonia intracellularis Decreases Shedding of Salmonella enterica serovar Typhimurium in Co-Infected Pigs and Alters the Gut Microbiome
Source: Sci Rep. 2018 Feb 12;8:2857. doi: 10.1038/s41598-018-21255-7 (PMC5809363; doi:10.1038/s41598-018-21255-7)
Supplement: Supplementary file 1 — Supplementary Information [file 41598_2018_21255_MOESM1_ESM.pdf]

Supplementary Information

Vaccination Against Lawsonia intracellularis Decreases Shedding of Salmonella enterica serovar Typhimurium in Co-Infected Pigs and Alters the Gut Microbiome

Fernando L. L. Leite[1], Randall S. Singer[1], Tonya Ward[2], Connie J. Gebhart[1], Richard E. Isaacson[1]*
1: Department of Veterinary and Biomedical Sciences, University of Minnesota, St. Paul, MN, USA
2: Biotechnology Institute, University of Minnesota, Minneapolis, MN, USA
*: Corresponding Author, isaac015@umn.edu

Supplementary Figure S1. Experimental outline. Treatment groups: Sal= *S.* Typhimurium challenged; Sal Law= *S.* Typhimurium, *L. intracellularis* co-challenge; Sal Law Vac= *S.* Typhimurium, *L. intracellularis* co-challenge and *L. intracellularis* vaccination; Sal Vac= *S.* Typhimurium challenge and *L. intracellularis* vaccination. Each box represents an isolation room with the number of animals per room.

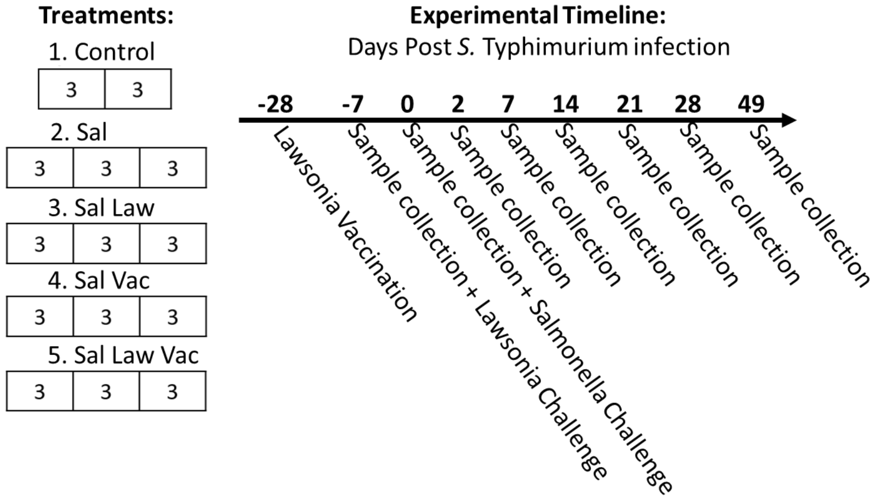

Supplementary Figure S2. Weighted UniFrac Distance between Control and Sal treatment groups at 0 a) and 14 days b) post *S.* Typhimurium challenge. Between is the distance between all samples in the comparison. Different letters indicate statistical significance (Wilcoxon rank-sum test $p<0.05$).

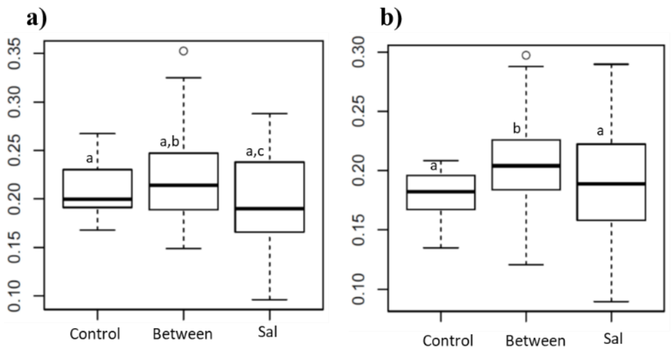

Supplementary Figure S3. Weighted UniFrac Distance between Control and Sal Law treatment group at 0 days post *S.* Typhimurium challenge (7 days post L. intracellularis challenge). Between is the distance between all samples in the comparison. Different letters indicate statistical significance (Wilcoxon rank-sum test $p<0.05$).

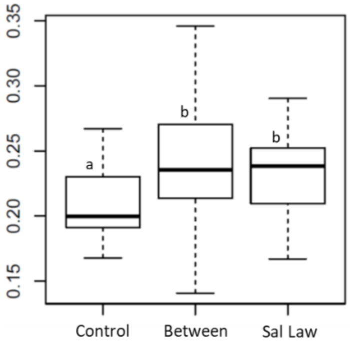

Supplementary Figure S4. Weighted UniFrac Distance between Sal Law and Sal Law Vac treatment group at 14 a) and 21 b) days post *S*. Typhimurium challenge. Between is the distance between all samples in the comparison. Different letters indicate statistical significance (Wilcoxon rank-sum test $p<0.05$).

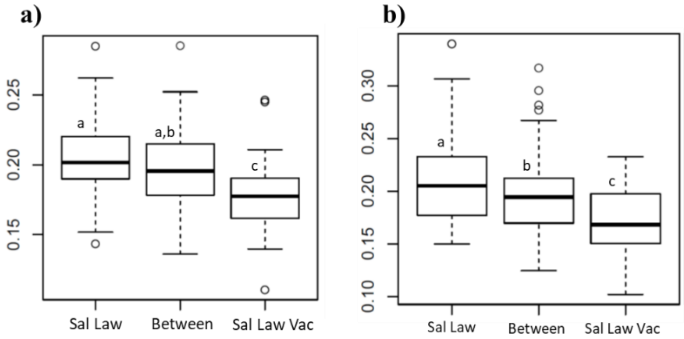

Supplementary Figure S5. Weighted UniFrac Distance between Sal and Sal Vac treatment group at 7 days post *S.* Typhimurium challenge. Between is the distance between all samples in the comparison. Different letters indicate statistical significance (Wilcoxon rank-sum test $p<0.05$).

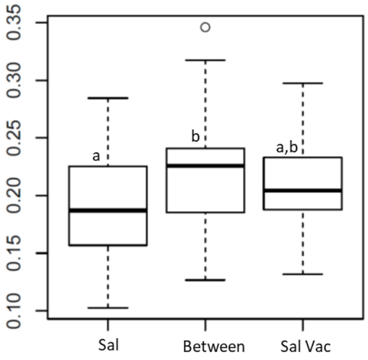

Supplementary Figure S6. Aplha diversity metrics of Chao 1 a) and number of observed OTUs b) at 7 days post *Salmonella enterica* serovar Typhimurium infection. No stastical significance (Wilcoxon rank-sum test *p*<0.05) observed between groups.

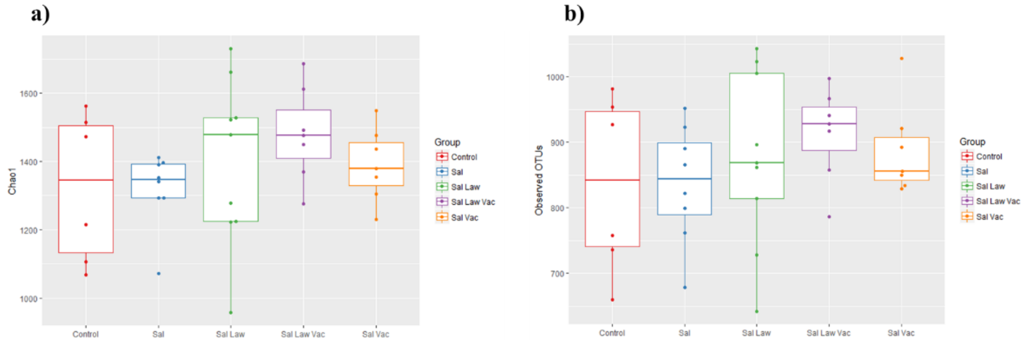

Supplementary Figure S7. Relative abundance of different phyla at 7 days post *Salmonella enterica* serovar Typhimurium infection.

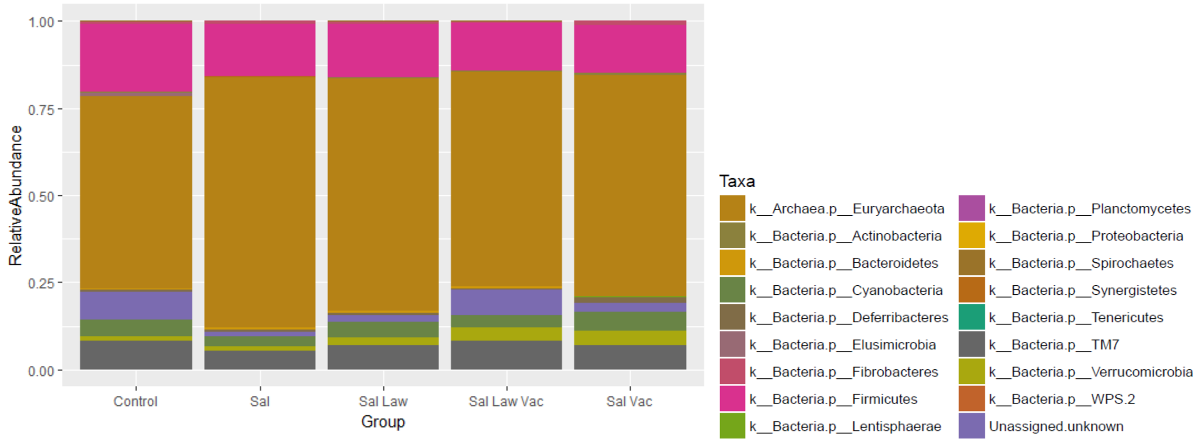

Supplementary Figure S8. Differentially abundant bacteria identified comparing different treatments at 0 days post *S.* Typhimurium infection. Each dot represents a different OTU identified within a genus. a) Control compared to Sal, a positive log fold change indicates enrichment in the Sal group (prior to challenge). b) Control compared to Sal Law, a positive log fold change indicates enrichment in the Sal Law group (only infected with *L. intracellularis* at this timepoint) ; c) Sal compared to Sal Law, a negative log fold change indicates decreased abundance in the Sal Law group (only infected with *L. intracellularis* at this timepoint); d) Sal Law compared Sal Law Vac, a positive log fold change indicates enrichment in the Sal Law Vac group associated with vaccination (only infected with *L. intracellularis* at this timepoint). * = OTU sequences closely related to *Clostridium butyricum*.

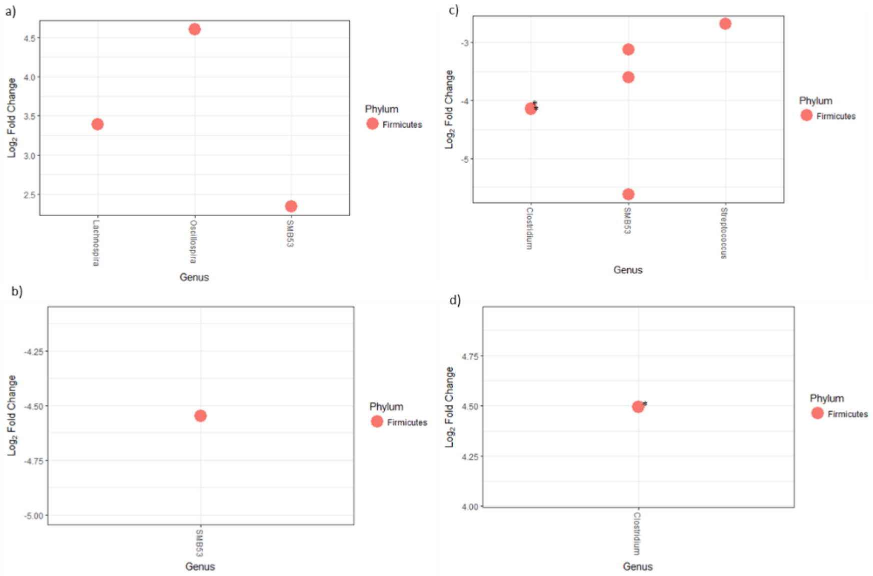

Supplementary Figure S9. Differentially abundant bacteria identified with *Lawsonia intracellularis* infection at day 0 prior to *S.* Typhimurium infection. The Sal Law group (only challenged with *L. intracellularis*) was compared to the Sal and Control groups (not challenged). A positive log fold change indicates enrichment with *L. intracellularis* infection. * = OTU sequences closely related to *Clostridium butyricum*.

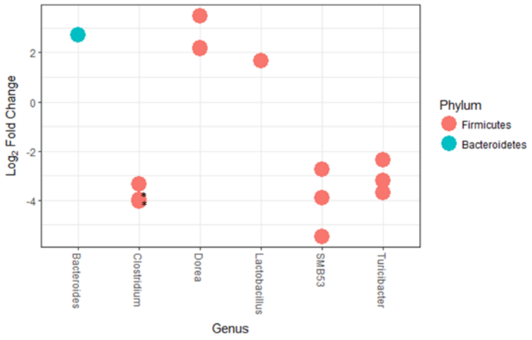

Supplementary Figure S10. Differentially abundant bacteria identified comparing different treatments at 7 days post *S.* Typhimurium infection. a) Sal compared to the Sal Vac group, a positive log fold change indicates enrichment with in the Sal Vac grup; b) Sal Law compared to the Sal Vac group, a positive log fold change indicates enrichment in the Sal Vac group. * = OTU sequences closely related to *Clostridium butyricum*. OTU of *Prevotella* genus did not match to a species level.

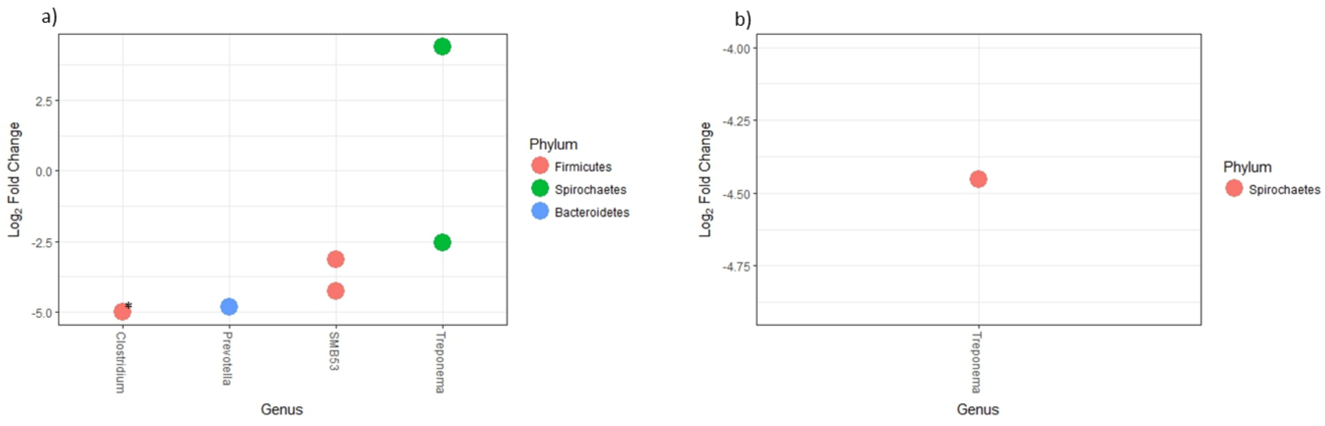

Supplementary Figure S11. The effect of live oral vaccination against *Lawsonia intracellularis* infection on the microbiome at timepoint 0 days post S. Typhimurium infection. The Sal Law group (only challenged with *L. intracellularis*) was compared to the Sal and Control groups at 0 days post *S.* Typhimurium infection. A positive log fold change indicates enrichment with vaccination. * = OTU sequences closely related to *Clostridium butyricum*. OTU of *Prevotella* genus did not match to a species level.

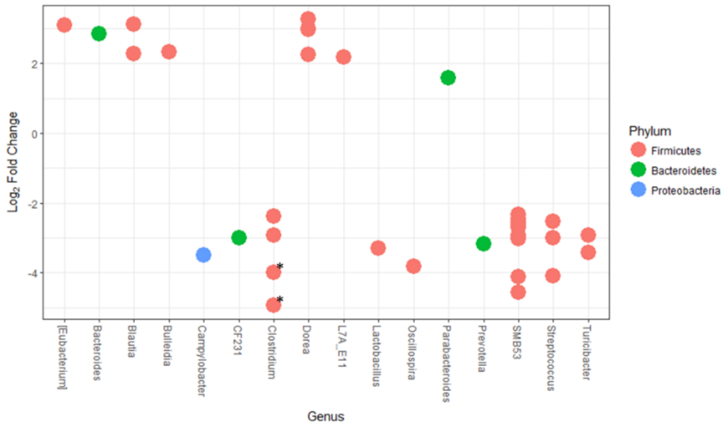

Supplementary Table S1. Description of operational taxonomic units (OTUs) mentioned in manuscript and their correlation to shedding level of *S.* Typhimurium at 7 days post infection.

OTU	Family	Genus	species	rho
541328	Clostridiaceae	Clostridium	butyricum	-0.4
4364745	Clostridiaceae	Clostridium	butyricum	
197994	Prevotellaceae	Prevotella	copri	0.51
198502	Prevotellaceae	Prevotella	copri	
527874	Prevotellaceae	Prevotella	copri	
denovo233554	Coriobacteriaceae	Collinsella	aerofaciens	0.44
189294	Coriobacteriaceae	Collinsella	aerofaciens	

rho= coeficient from Spearman's rank correlation between relative abundance of OTUs and shedding of *S.* Typhimurium at 7 days post infection, *p* values of three coeficients < 0.05.
